# Supplementary material for: Electrospun Nanofiber Covered Polystyrene Micro-Nano Hybrid Structures for Triboelectric Nanogenerator and Supercapacitor
Source: Micromachines (Basel). 2022 Feb 26;13(3):380. doi: 10.3390/mi13030380 (PMC8951335; doi:10.3390/mi13030380)
Supplement: Supplementary file 1 [file micromachines-13-00380-s001.zip › micromachines-1614702-supplementary.pdf]

micromachines

Supplementary Materials: Electrospun Nanofiber Covered Polystyrene Micro-Nano Hybrid Structures for Triboelectric Nanogenerator and Supercapacitor

Jihyeon Park [1,2], Seungju Jo [1,2], Youngsu Kim [1,2], Shakir Zaman [1,2] and Daewon Kim [2,3,*]

1 Department of Electronics and Information Convergence Engineering, Kyung Hee University, 1732 Deogyeong-daero, Giheung-gu, Yongin 17104, Korea; jihyeon.park@khu.ac.kr (J.P.); joseungju@khu.ac.kr (S.J.); youngsukim@khu.ac.kr (Y.K.); shakirzaman@khu.ac.kr (S.Z.)
2 Institute for Wearable Convergence Electronics, Kyung Hee University, 1732 Deogyeong-daero, Giheung-gu, Yongin 17104, Korea
3 Department of Electronic Engineering, Kyung Hee University, 1732 Deogyeong-daero, Giheung-gu, Yongin 17104, Korea
* Correspondence: daewon@khu.ac.kr

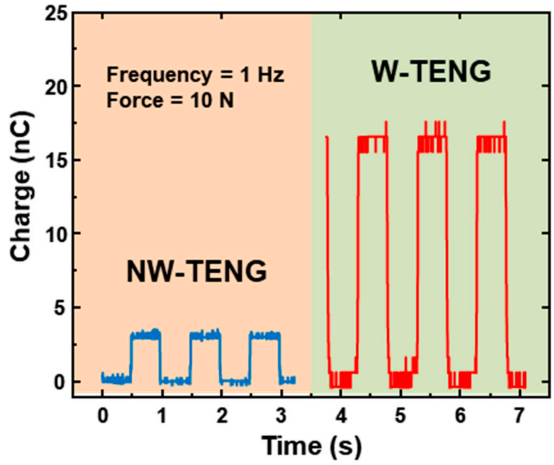

Figure S1. The amount of transferred charge from the NW-TENG and W-TENG.

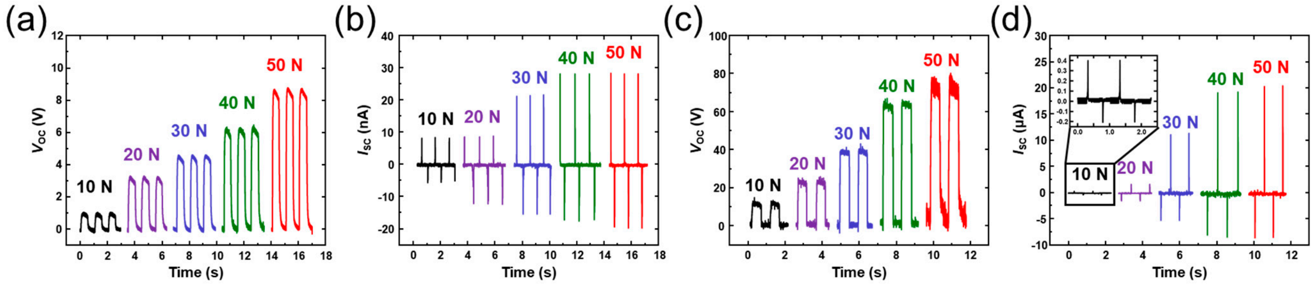

Figure S2. (a) Output voltage and (b) current of the NW-TENG at different applied force from 10 N to 50 N at constant applied frequency of 1 Hz. (c) Output voltage and (d) current of the W-TENG at different applied force from 10 N to 50 N at constant applied frequency of 1 Hz.

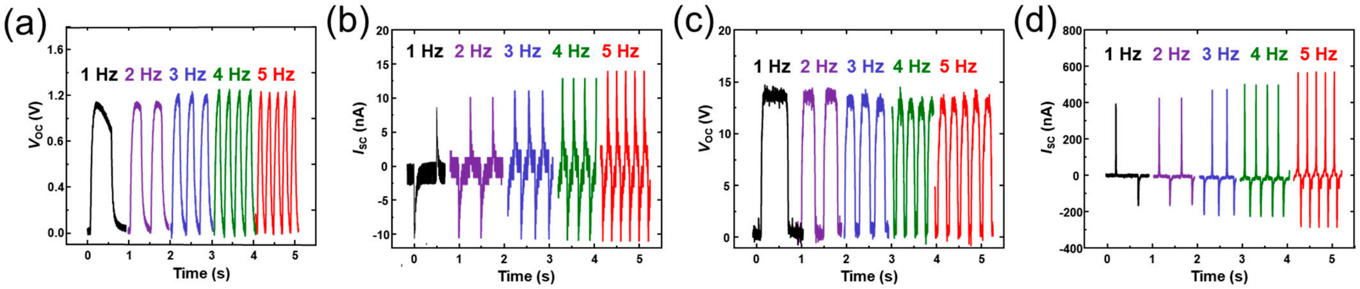

Figure S3. (a) Output voltage and (b) current of the NW-TENG at different applied frequency from 1 Hz to 5 Hz at constant applied force of 10 N. (c) Output voltage and (d) current of the W-TENG at different frequency from 1 Hz to 5 Hz at constant applied force of 10 N.

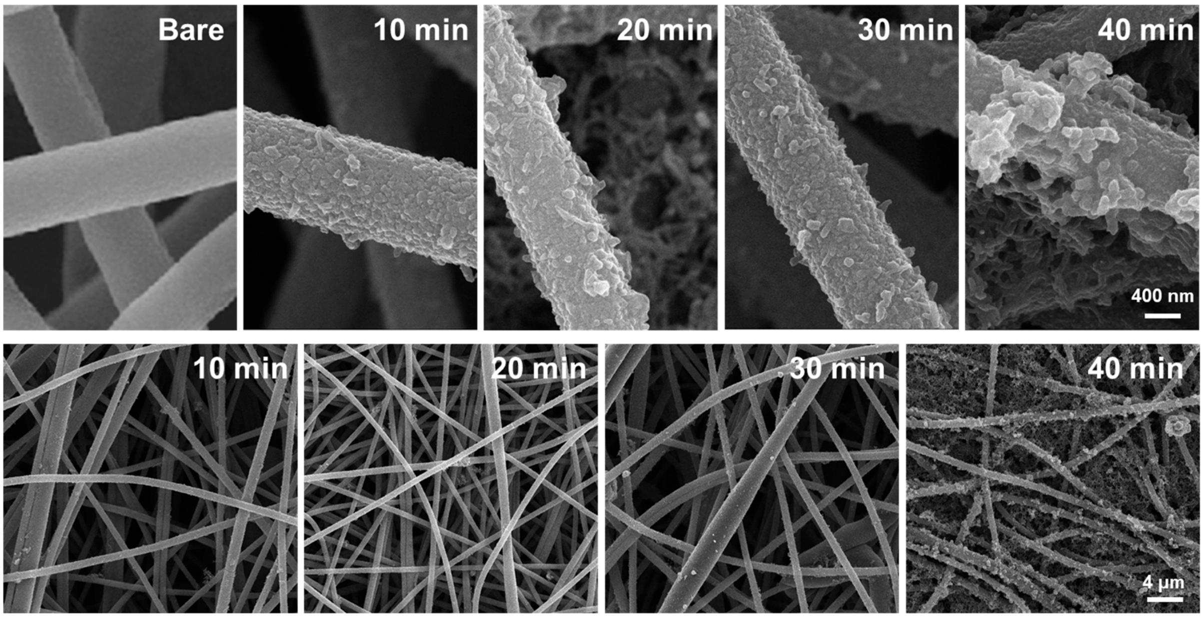

Figure S4. FE-SEM image of PANI coated PAN membrane at different coating times.
